# The Multifaceted Neurotoxicity of Astrocytes in Ageing and Age-Related Neurodegenerative Diseases: A Translational Perspective

**DOI:** 10.3389/fphys.2022.814889

**Published:** 2022-03-17

**Authors:** David S. Bouvier, Sonja Fixemer, Tony Heurtaux, Félicia Jeannelle, Katrin B. M. Frauenknecht, Michel Mittelbronn

**Affiliations:** ^1^National Center of Pathology (NCP), Laboratoire National de Santé (LNS), Dudelange, Luxembourg; ^2^Luxembourg Center of Systems Biomedicine (LCSB), University of Luxembourg (UL), Belvaux, Luxembourg; ^3^Luxembourg Center of Neuropathology (LCNP), Dudelange, Luxembourg; ^4^Systems Biology Group, Department of Life Sciences and Medicine (DLSM), University of Luxembourg, Belvaux, Luxembourg; ^5^Institute of Neuropathology, Medical Center of the Johannes Gutenberg University Mainz, Mainz, Germany; ^6^Department of Cancer Research (DOCR), Luxembourg Institute of Health (LIH), Luxembourg, Luxembourg; ^7^Faculty of Science, Technology, and Medicine (FSTM), University of Luxembourg, Esch-sur-Alzette, Luxembourg; ^8^Department of Life Sciences and Medicine (DLSM), University of Luxembourg, Esch-sur-Alzette, Luxembourg

**Keywords:** astrocyte, neurotoxicity, reactive astrogliosis, heterogeneity, neurodegeneration, ageing

## Abstract

In a healthy physiological context, astrocytes are multitasking cells contributing to central nervous system (CNS) homeostasis, defense, and immunity. In cell culture or rodent models of age-related neurodegenerative diseases (NDDs), such as Alzheimer’s disease (AD) and Parkinson’s disease (PD), numerous studies have shown that astrocytes can adopt neurotoxic phenotypes that could enhance disease progression. Chronic inflammatory responses, oxidative stress, unbalanced phagocytosis, or alteration of their core physiological roles are the main manifestations of their detrimental states. However, if astrocytes are directly involved in brain deterioration by exerting neurotoxic functions in patients with NDDs is still controversial. The large spectrum of NDDs, with often overlapping pathologies, and the technical challenges associated with the study of human brain samples complexify the analysis of astrocyte involvement in specific neurodegenerative cascades. With this review, we aim to provide a translational overview about the multi-facets of astrocyte neurotoxicity ranging from *in vitro* findings over mouse and human cell-based studies to rodent NDDs research and finally evidence from patient-related research. We also discuss the role of ageing in astrocytes encompassing changes in physiology and response to pathologic stimuli and how this may prime detrimental responses in NDDs. To conclude, we discuss how potentially therapeutic strategies could be adopted to alleviate or reverse astrocytic toxicity and their potential to impact neurodegeneration and dementia progression in patients.

## Introduction

### Designed to Protect, Maintain, Support, and Regulate Brain Function

The paradigm on glial cells has considerably shifted over the last 20 years. Glial cells are now recognized to be at the center of many primordial processes for brain homeostasis maintenance but also involved in protective as well as, paradoxically, detrimental responses. Astrocytes represent the most abundant glial cell type of the CNS. They form a heterogeneous group of cells, comprising distinct subtypes characterized by a specific morphology, physiology, or spatial distribution (Pestana et al., 2020). The general classification distinguishes mainly white matter fibrous astrocytes from grey matter protoplasmic astrocytes. However, numerous specialized subtypes have been described in distinct brain areas such as the Bergman glia in the cerebellum (De Zeeuw and Hoogland, 2015), the Müller glia in the retina, or the interlaminar astrocytes in the neo-cortex (Colombo, 2018) or in specific localization such as the perivascular astrocytes or the subpial astrocytes. The morphology, density, overlap, and diversity of astrocytes can vary depending on brain areas and species (Oberheim et al., 2009; Batiuk et al., 2020; Muñoz et al., 2021). The classic description of the astrocyte, representing mainly protoplasmic ones, is the following:

- A complex morphology made by long processes and protrusions (Bushong et al., 2004), sculpted around a “star-shaped” cytoskeleton frequently highlighted by the staining of the intermediate filament, the glial fibrillar acidic protein (GFAP).
- An exclusive parenchyma territory with peripheral overlapping cellular contacts to neighboring astrocytes by gap-junctions such as connexins 30 and 43 (Bushong et al., 2002; Huang et al., 2021).
- Specialized compartments, the endfeet, which enwrap blood vessels and take-up nutrients from the circulation.
- Microcompartments enveloping pre- and post-synapses, forming together a tripartite synapse, with an estimation of 10^5^–10^6^ synapses contacted/enwrapped per astrocyte (Volterra et al., 2014; Allen and Eroglu, 2017).

Many of these features considerably differ between the astrocyte subtypes. The interlaminar astrocytes, located in the first layer of some of the neocortical areas, project long processes with varicosities in deeper layers. Twin astrocytes are joined by their soma, and perivascular astrocytes have their cell bodies sitting on blood vessels (Verkhratsky and Nedergaard, 2018). Still, astrocytes are multitasking cells being responsible for the metabolic support of neurons by taking up glucose by their privileged connection to the blood flow, delivering it to the surrounding cells and storing glycogen in the CNS. They also serve as extracellular milieu buffering cells (K^+^, Cl^−^, Ca^2+^, and water). Many reviews have extensively discussed the key roles of astrocytes in brain metabolism (Deitmer et al., 2019; Rose et al., 2020). At the synapse, astrocytes act as sensors and modulators of synaptic activity. They express the glutamate transporters EAAT1 (GLAST) and EAAT2 (GLT1) enriched in perisynaptic processes. It allows them to pump the excess of neurotransmitters at the synaptic cleft and recycle them. However, astrocytes can also directly release neurotransmitters, such as glutamate, D-serine, and/or ATP and modulate synaptic activity (Araque et al., 2014; Volterra et al., 2014). The neuron-astrocyte communication is bilateral and fundamental for brain function. Astrocytes are intimately associated with the establishment and maintenance of neuronal circuits. During development, they remodel the extracellular matrix by secreting some matricellular proteins, such as secreted protein acidic and rich in cysteine (SPARC), Tenascin C, or/and Thrombospondins (Jones and Bouvier, 2014). Astrocytes also eliminate unnecessary excitatory synapses through MEGF10 and MERKT phagocytosis receptors (Chung et al., 2013). Their multiple functions at the cellular level are commonly accepted, however, there are still pending questions about their role as a collective entity in higher brain functions. Indeed, they form a widespread and dynamic network of non-excitable cells, communicate *via* calcium transient waves, and provide an active cell layer for information and modulation of CNS homeostasis (Guerra-Gomes et al., 2018).

### Being Reactive Is Not Being Toxic?

Astrocytes are also directly involved in CNS protection from pathogens and pathologies. They are highly adaptive to their micro-environment, and in adverse conditions, they can change their molecular program and morphology to counteract insults and protect surrounding tissue. This phenomenon is called reactive astrogliosis (see consensus statement in Escartin et al., 2021). Reactive astrocytes are commonly observed in virtually all brain disorders. The shift of morphology toward large and dysmorphic processes, and the increase of the expression of proteins forming the intermediate filaments, GFAP and vimentin, are often considered as the main characteristics to characterize reactive astrogliosis. But the reactivity of astrocytes is neither an “all or none” nor a unidirectional mechanism. It is now described as a gradient of severity from mild to severe with a contextual time course (Sofroniew and Vinters, 2010). Their reactivity is also heterogeneous, conditioned by their intrinsic nature (species, localization, age, gender, genetics background, and epigenetics), their macro-environment (stage of a disease, brain region vulnerability) and the immediate pathological micro-environment. By becoming reactive, an astrocyte can gain or lose some functions, alter its physiology in the short- or long-term, modify its interplay with surrounding cells, or even engage detrimental responses. Reactive astrocytes can produce growth factors and neurotrophins, thereby promoting neuronal survival and synaptic function (Sofroniew and Vinters, 2010; Escartin et al., 2021). They can mimic immune responses and secrete a large variety of anti- and pro-inflammatory molecules, such as interferon gamma (IFN-γ), tumor necrosis factor alpha (TNF-α), interleukin (IL)-6 and IL-1β or act as antigen-presenting cells. Although many of those inflammation-related factors are mainly expressed by cells of the myeloid lineage, we here focused on the potential capacity of astrocytes to also secrete those molecules under pathological conditions. Depending on the context, they work in synergy or interfere with microglia or with infiltrating immune cells. However, in numerous models, they can also alter the surrounding cells by secreting toxic factors and drive the progression of neurodegeneration. The questions about where and when astrocytes lose their protective functions and become toxic for surrounding cells are fundamental to solve the complex puzzle of brain disorders and age-related neurodegenerative diseases (NDDs), such as Alzheimer’s disease (AD), Parkinson’s disease (PD), Lewy body dementias (LBDs), primary tauopathies, primary synucleinopathies, fronto-temporal dementia (FTD), and amyotrophic lateral sclerosis (ALS).

This review is composed of two main parts. The first chapter is about how, when and why astrocytes turn neurotoxic: from the attempts to define a prototypical neurotoxic molecular signature across various disease models, to the characterization of stressors associated with NDDs that have been shown to induce astrocyte neurotoxicity and subsequent damages or death of neurons in culture and rodent models. We then compile reports about the identification of neurotoxic astrocytic markers in autopsy brain samples from NDDs patients. In the second chapter, we discuss the impact of ageing on astrocytes, on their senescence and epigenetics and if the ageing process could prime them to execute maladaptive/toxic responses in NDDs. Finally, we discuss the challenges targeting reactive/neurotoxic/aged/senescent astrocytes to alleviate NDDs progression.

## Breaking Bad: When do Astrocytes Become Toxic to Surrounding Cells in NDDs?

### The Neurotoxic Astrocyte Signatures: Identity and Context of Harmful Astrocytes

Because astrocytes are at the core of brain homeostasis and function, their turnover into neurotoxic cells could trigger or exacerbate NDDs. Thus, it is essential to precisely characterize their changes in NDDs. Astrocytes are often found to be atrophic or dysmorphic in AD and associated dementias (see the chapter “Pieces of Evidence of Astrocyte Neurotoxicity in NDD Patient Samples”). However, to ponder on the dual faces of astrocytes, it is important to distinguish between reactivity and toxicity, between chronic changes and acute responses. The reactivity state engages various molecular and morphological changes (Viejo et al., 2021), and is a direct consequence of alterations of their macro- and/or microenvironment (Sofroniew and Vinters, 2010; Haim et al., 2015; Escartin et al., 2021). Chun et al. (2020) designed a new mouse model to modulate stages of astrocyte reactivity, from mild to severe, by crossing inducible diphtheria toxin receptor (iDTR) mice with GFAP-CreERT2 mice (GiD). The severity of the reactivity in GiD mice has been scaled by the level of GFAP expression, the dystrophy and branching of processes, by some proliferation and astrocytic production of monoamine oxidase B (MAO-B), GABA, and inducible nitric oxide synthase (iNOS). The activation of severe reactive profiles led to pronounced atrophy, particularly in the CA1 subregion of the hippocampus, but also in the cortex, striatum, and amygdala, and an increase in cleaved caspase-3 expression as well as tauopathy in neurons. Behavioral tests on severe GiD mice also showed memory impairments. Thus, severe astrocyte reactivity is neurotoxic and can trigger, if induced systematically, some NDDs features and symptoms. The authors also observed a gradient of severity across the brain.

Using single-cell RNA sequencing (scRNAseq) and spatial transcriptomics, Hasel and colleagues characterized ten astrocytic clusters with specific molecular signatures across the mouse brain in responses to systemic lipopolysaccharide (LPS) treatment (Hasel et al., 2021). Among these clusters, that also changed over time post-injection, none of them was characterized as fully neurotoxic. Inflammatory genes increased in some clusters and were found along with potentially neuroprotective genes in others, highlighting the complexity of astrocyte responses. Numerous studies have attempted to define a prototypical molecular signature for neurotoxic astrocytes. *In vitro*, with isolated astrocytes from young adult (P30-P35) transgenic Aldh1l1-eGFP mice, Zamanian et al. (2012) observed various astrocytic reactive responses dependent on the type of perturbation. The authors described that reactive astrocytes isolated from the cortex, corpus callosum, hippocampus, and striatum of mice with ischemic stroke showed a particular “protective” profile with induced neurotrophic cytokines LIF and CLCF1, IL-6, and some other genes related to metabolic activity, cell-cycle genes, and transcription factors. On the other hand, the reactive astrocytes isolated from the cortex and corpus callosum from mice that underwent systemic LPS treatment presented what was described as a “detrimental” profile with an increased expression of genes related to the induced antigen presentation pathway, complement pathway, interferon response, class I major histocompatibility complex (MHC) molecules (H2-D1, H2-K1, and H2-T10), and complement cascade (initiating: C1r, C1s, C3, and C4B; inhibiting: Serping 1). Both types of astrocytes share a set of upregulated genes, including proteins involved in extracellular matrix modification and cytokine signaling. Liddelow and colleagues gave a more detailed description of the reactive signatures obtained in these models (Liddelow et al., 2017). They defined two signature groups of reactive astrocytes. The neurotoxic one is now named A1, the neuroprotective A2, both sharing a pan-reactive astrocyte set of increased genes. In their co-culture model, A1 astrocytes decreased synaptogenesis and even induced neuronal death at high concentrations. Furthermore, the authors demonstrated that the A1 neurotoxic signature was dependent on the microglia-released pro-inflammatory cytokines (see the section “Interplay Between Astrocytes and Immune Cells: the Control of Neurotoxicity?”). The authors choose C3 as a marker for A1 astrocytes and identified C3-positive astrocytes in human post-mortem brain tissue from AD, PD, ALS, multiple sclerosis (MS), and Huntington disease (HD). Since then, many studies using NDDs models have reported at least a partial A1 neurotoxic signature in their results (see the section “Maladaptive Responses to Stressors in Cell Culture and Transgenic Rodents Modeling NDDs”), but there is no consensus that this signature is generally found in NDDs. Other reports have added some insights in distinct astrocytic neurotoxic molecular signatures in NDDs.

Smith et al. (2020) have investigated the role of the unfolded protein response (UPR), a pathway dysregulated in NDDs, in the response of astrocytes. Using the endoplasmic reticulum (ER) stressors thapsigargin or tunicamycin, they have chronically activated the UPR through protein kinase R-like ER kinase (PERK) pathways in cortical primary astrocytes (Smith et al., 2020). The authors observed an upregulation of the pan-reactive markers Cxcl10, Lipocalin 2 (Lcn2), and Vimentin (Vim) upon thapsigargin treatment. C3 was the only gene increased of the A1 signature, but Ggta1 and Serping1 were significantly reduced. The A2 markers Cd109, Emp1 were also significantly decreased. Blocking PERK activation through a knockdown let to deceased expression of C3, Cxcl10, Lcn2, and Vim. The authors reproduced these data in a prion-diseased mouse and demonstrated *in vitro* that the UPR-reactive astrocytes presented an altered secretome, were unable to support synapses and harmful to neurons.

Wheeler et al. (2020) performed scRNAseq on brain and spinal cord from experimental autoimmune encephalomyelitis (EAE) mice and fresh autopsy brain samples from MS patients. In EAE mice, the largest subgroup of astrocytes was enriched for UPR, showing high level of NF-κB and iNOS pathways activation, as well as granulocyte-macrophage colony-stimulating factor (GM-CSF) signaling. In the same cluster, the authors also identified transcriptional regulators including Kdm5a, Hif1a, Fos, and Jun and to a lower extent Nfe2l2. Nfe2l2 encodes the transcription factor NRF2 (nuclear factor erythroid 2-related factor 2), a limiter for oxidative stress and inflammation. The authors further confirmed with *in vitro* experiments that NRF2 is a negative regulator of pro-inflammatory and neurotoxic pathways. Some markers of the A1 signature were found throughout the different clusters isolated (Cluster 0: H2-T23, cluster 1: H2-D1, Psmb8, cluster 2: Srgn, and cluster 4: C3ar1). To identify astrocyte regulators, the authors have isolated astrocytes from Ribotag^Gfap^ mice during EAE. They have observed increased levels of the small MAF protein musculoaponeurotic fibrosarcoma homolog G (MAFG), concomitant with a decreased expression of NRF2. After analysis of their scRNAseq data from fresh autopsy MS brain tissue and previous published MS data (Lake et al., 2018; Jäkel et al., 2019; Schirmer et al., 2019), they have recovered an astrocyte population with the same molecular signature. At the tissue level, the authors observed MAFG-positive astrocytes in active lesions of white matter from MS patients. They have concluded that MAFG-positive astrocytes are harmful and promote CNS inflammation in EAE and MS.

Habib et al. (2020) performed single-nucleus RNAseq (snRNAseq) analysis on hippocampi from 5xFAD mice. Compared to WT, the AD mice displayed an enriched astrocytic cluster expressing high levels of Gfap, Serpina3n, Ctsb, Apoe, and Clu17, which they termed disease-associated astrocytes (DAAs). The authors found an overlap of some A1 genes in the DAAs. A2 signature was not seen. Interestingly, an increase of A1/DAAs astrocytes was associated with ageing in WT mice. The authors were also able to retrace DAA-like cells in another snRNAseq database from the post-mortem human AD prefrontal cortex (Mathys et al., 2019). Zhou et al. (2020b) also performed snRNAseq on 5xFAD mice and dorsolateral pre-frontal cortex from AD patients with R62H variant of TREM2. In 5XFAD mice, astrocytes displayed an upregulation of Gfap and C4b. Interestingly they observed an upregulation of Serpina3n, previously linked to DAAs, mainly in oligodendrocytes. They also detected little colocalization of Serpina3n with astrocytes and amyloid-β plaques. In human AD samples, they observed a downregulation of a cluster that was highly enriched in genes controlling free-fatty acid transport (FABP5), storage in lipid droplets (HILPDA), as well as oxidation and detoxification of the resulting reactive oxygen species (ROS; SOD2). On the other hand, AD astrocytes presented an increase in the expression of genes encoding the proteoglycan NCAN and collagen COL5A3. The authors speculated that these extracellular matrix molecules may contribute to glial scarring and could prevent axonal regeneration. The authors could not identify A1 signatures.

**Figure 1:**
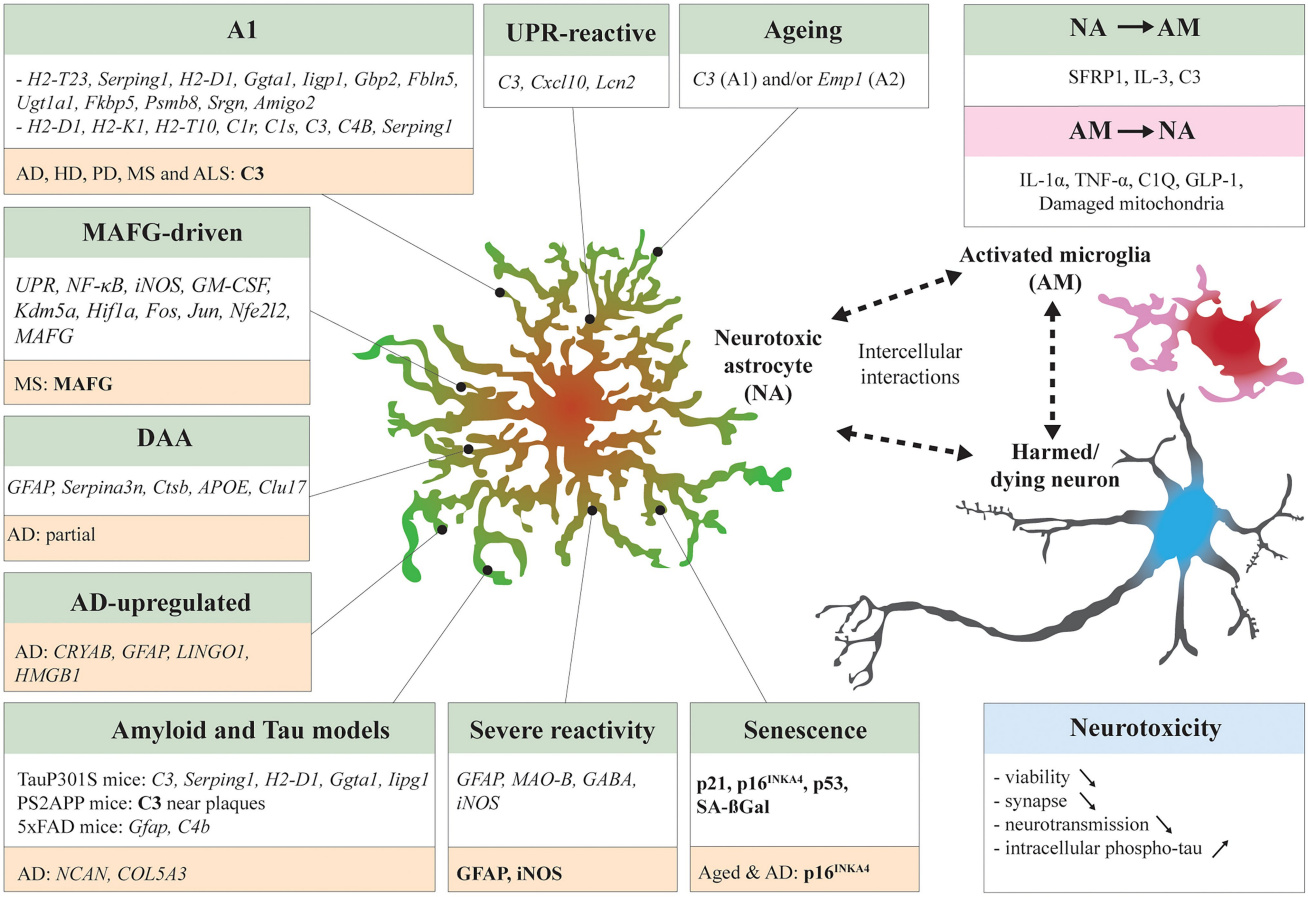
Neurotoxic astrocytic signatures and interaction with neighboring cells. Many studies have identified neurotoxic astrocytic molecular signatures (italic) and markers (bold) across specific experimental conditions: A1 in systemic LPS treated mice (Zamanian et al., 2012; Liddelow et al., 2017), unfolded protein response (UPR)-reactive astrocytes in UPR-activated mice and prion-diseased mice (Smith et al., 2020), musculoaponeurotic fibrosarcoma homolog G (MAFG)-driven astrocytes in experimental autoimmune encephalomyelitis (EAE) mice (Wheeler et al., 2020), Disease associated astrocytes (DAAs) in 5xFAD mice (Habib et al., 2020), Alzheimer’s disease (AD)-upregulated astrocytes in AD patients (Lau et al., 2020), Aged mouse astrocytes (Clarke et al., 2018), senescent mouse astrocytes (Bussian et al., 2018; Gaikwad et al., 2021), and severe reactive mouse astrocytes (Chun et al., 2020). Other studies have compared neurotoxic astrocyte signatures in distinct disease mouse models such as TauP301S mice and PS2APP mice (Wu et al., 2019) and 5xFAD mice (Zhou et al., 2020b). Some signatures overlapped between models. In NDDs patient samples, neurotoxic astrocyte markers have been identified by transcriptomics or IHC (highlighted in orange). The neurotoxic astrocytes are in close bilateral communication with surrounding cells. Activated microglia can induce astrocytes to adapt a neurotoxic profile (Liddelow et al., 2017; Joshi et al., 2019; Park et al., 2021), vice versa neurotoxic astrocytes secrete microglia-activating factors (McAlpine et al., 2021; Rueda-Carrasco et al., 2021). Finally, the induced neurotoxicity can decrease neuron viability, exacerbate synaptic loss, neurotransmission impairment, and tau pathology. Reciprocally, harmed or dying neurons can stimulate activation of microglia and reactivity of astrocytes (not detailed here).

The neurotoxic astrocytic signatures collected across models share few markers, but all play a role in the disease progression in their respective models. Some of these signatures were at least partially found in the brain tissue of patients (see the section “Pieces of Evidence of Astrocyte Neurotoxicity in NDD Patient Samples” for additional insights). However, these studies accentuate the idea of subgroups of astrocytes that become harmful, with a specific distribution or linked to a condition (resumed in Figure 1). In the following chapters, we will navigate between *in vitro* and mouse models to identify stressors that could alter astrocyte responses toward a detrimental role and thus recapitulate if astrocyte neurotoxicity has been detected and measured in brains from NDDs patients.

### Maladaptive Responses to Stressors in Cell Culture and Transgenic Rodents Modeling NDDs

In age-related NDDs, astrocytes, among other brain cells, are exposed to a variety of stressors. AD, PD, LBD, primary tauopathies, primary synucleinopathies, FTD, and ALS are often grouped under the umbrella appellation “proteinopathies” because they are characterized by abnormal accumulations of peptides or proteins in the extracellular milieu or inside brain cells. These accumulations are often thought to be the cause of NDDs and responsible for a complex chain of degenerative events. Proteinopathies are defined by one type or overlap of pathological misfolded protein inclusions (Golde et al., 2013). Overproduction and release in the extracellular space of the amyloid-β (Aβ) peptide 1–42 results in the formation of senile plaques in the parenchyma. The hyperphosphorylated form of tau, a microtubule-associated protein, is prone to cluster into paired helical filaments (PHF) and neurofibrillary tangles (NFTs), which are most often found inside neurons. Intracellular inclusions of phosphorylated α-synuclein (α-Syn) form Lewy neurites and Lewy bodies (LBs) in the soma of neurons. The accumulation of TAR DNA-binding protein 43 (TDP-43), a protein involved in DNA transcription and RNA modulation, is found in numerous cell types but primarily in neurons. The concomitance of these misfolded protein accumulations is common across NDDs. However, Aβ and tau pathologies *in vitro* and mouse models are usually considered to recapitulate AD, alpha-synuclein pathology, PD and TDP-43, and ALS.

The effect of the monomers, oligomers, or filaments of these proteins/peptides can be mimicked *in vitro*, and the parenchymal protein inclusions found in patients *in vivo* with transgenic mouse models expressing mutated forms of human genes. Astrocytes responses to such exposures have been well documented. Astrocytes have been involved in clearance, spreading, and propagation of Aβ, tau, and α-Syn pathologies (Batarseh et al., 2016; De Strooper and Karran, 2016; Frost and Li, 2017; Sorrentino et al., 2019). This literature is quite extended; therefore, we emphasize studies that found a direct association between pathological protein exposure and astrocytic neurotoxicity. We further highlight the “context” of the experimental procedures *in vitro*, characteristics of the model, age, brain regions, and nature of the transgene. All these aspects must be carefully examined before extrapolating it to the human situation, even more, when contradictive results have been published across these models.

#### Amyloid Pathology

Amyloid-ß pathology is central to AD progression. Many reports have shown a direct effect of Aβ exposure on the phenotype of astrocytes toward neurotoxic profiles in culture. Exposure to aggregated Aβ-42 peptides or oligomers induced, in a dose-dependent manner, the production of reactive oxygen species (ROS) and iNOS by cortical rat astrocytes through the activation of NF-κB pathways (Akama et al., 1998) and by human primary astrocytes (Singh et al., 2020). It was shown that even picomolar concentrations of Aβ-40 and Aβ-42 oligomers destabilized calcium activity in rat hippocampal astrocytes in co-culture with neurons, increase their ROS production and caspase-3 activation in both astrocytes and neurons (Narayan et al., 2014). *In vitro*, astrocytes seem to mediate Aβ-induced toxicity on neurons. Indeed, Garwood et al. (2011) have shown that the viability of cortical neurons was not perturbed by oligomeric Aβ at their working concentration but was impacted by altered astrocytes in mixed cultures. In this model, Aβ-exposed astrocytes increased cleaved-caspase 3 induction, tau truncation, and phosphorylation in neurons. This deleterious effect was rescued by minocycline, which dampens the secretion of inflammatory factors, such as IL-6, IL-1β, and IFN-γ of Aβ-exposed astrocytes. Numerous markers of astrocytic toxicity have also been found in transgenic rodent modeling the amyloid pathology, where the intensity of astrocytic reactivity usually correlates with pathology (Spanos and Liddelow, 2020). Amyloid transgenic rodents express a mutated form of the human amyloid precursor protein (APP) or/and presenilin, thus mimicking the early onset familial form of AD, and the formation of Aβ plaques in the parenchyma. These models recapitulate numerous features of AD such as synaptic loss or cognitive defect but no to only poor neuronal loss. Thus, the active role of neurotoxic astrocytes in the atrophy processes cannot be considered in this context. However, the astrocytes surrounding the plaques, that form the reactive glial net (RGN) together with microglia, have been particularly studied (Bouvier et al., 2016; Walker, 2020). RGN astrocytes can express inflammatory factors, such as IL-6 or IL-1β in CRND8Tg (Bouvier et al., 2016), iNOS in APP (V717I; Heneka et al., 2005), and C3 in 3xTg, Tg2576, and PS2APP mice (Fonseca et al., 2011; Wu et al., 2019) and TgF344-AD rats (Balu et al., 2019).

Interestingly, the control or ablation of astrocyte reactivity in amyloid models had different outcomes depending on the experimental strategy and the model used. Kraft et al. (2013) designed an APP/PS1 Gfap^−/-^Vim^−/−^ model to dampen astrocyte hypertrophy and reactivity in amyloidosis conditions. It resulted in a large increase in plaque load, of the number of microglia associated with plaques and of the neurites dystrophia at 8 and 12 months of age, arguing for a beneficial impact of astrocyte reactivity in AD. The inducible ablation of proliferative reactive astrocytes in APP/GFAP-TK mice, treated with ganciclovir at 9 months of age, significantly increased the levels of monomeric Aβ-42 and exacerbated synaptic loss, neuroinflammation, and memory deficits (Katsouri et al., 2020). The selective pharmacological ablation of astrocytes with the toxin L-alpha-aminoadipate in organotypic brain culture slices (OBCSs) from 7 days old 5xFAD animals, grown over 2 weeks, led to an increase of Aβ levels in medium, of IL-6 production, and decrease in spine size (Davis et al., 2020). All these results favor a protective role of reactive astrocytes in disease progression, however, other studies have reported opposite conclusions. Furman and collaborators used a different methodological approach that consists of injecting a viral construct into APP/PS1 mice hippocampi to selectively express a synthetic peptide named VIVIT in astrocytes, which will inhibit their inflammatory response (Furman et al., 2012). The authors found that treated 7–8-month-old mice had significantly lower soluble and insoluble amyloid levels, reduced microglia activation, and improved cognitive performance at 16–17 months of age, compared to the non-treated transgenic mice. Other studies highlighted the pathological and detrimental roles of astrocytes in amyloid models of AD. Two different strategies to inhibit the Janus kinases (JAK)/signal transducer and activator of transcription 3 (STAT3) pathway, which is deeply involved in the induction of the reactivity of astrocytes, led to similar conclusions. Ceyzériat et al. (2018) used viral construction to overexpress an inhibitor of JAK, the suppressor of cytokine signaling 3 (SOCS3) in the CA1 hippocampal astrocytes of 3–4-month-old APP/PS1dE9 mice. Six months after injection, they reported a downregulation of the pro-inflammatory responses in transfected astrocytes, a reduction of plaque loads, and improved spatial learning compared to non-transfected transgenic. After SOCS3 transfection in astrocytes of 3xTg, the authors described a complete restoration of long-term potentiation (LTP) deficits. Reichenbach et al. (2019) have further investigated the impact of astrocytic Stat3 induced pathway in an inducible Stat3 deficient APP/PS1 mouse model. When the Stat3 knock-out in astrocytes was initiated at 6 weeks of age, effects were beneficial showing a decrease of amyloid loads, neuronal dystrophy, cytokines levels, and rescue of memory decline at 8–10 months of age. The A1 transcripts Amigo2 and C3 were significantly decreased compared to age-matched APP/PS1.

In conclusion, in most models *in vitro* and *in vivo*, astrocytes engage neurotoxic responses that could exacerbate disease progression. However, there is no consensus and neither a prototypical neurotoxic signature.

#### Tau Pathology

The tau pathology is often described in neurons, which are mainly bearing PHF and NFT in the AD patient brain. However, tau positive astrocytes are also found in primary tauopathies and less often in AD (see the section “Pieces of Evidence of Astrocyte Neurotoxicity in NDD Patient Samples”). The relationship between tau and the neurotoxicity of astrocytes is still not clearly defined. However, the exposure of monomeric tau or tau fibrils on primary astrocytes led to their internalization and subsequent integrin- and NF-κB-dependent production of neurotoxic factors, among them typical cytokines, such as IL-6, IL-1β, TNF-α, and CCL10, and a consecutive decrease of neuronal viability in culture (Wang and Ye, 2021). In this study, the molecular profiling of phosphorylated-tau-exposed astrocytes revealed many similarities with the A1 signature, Gibp2, and Ligp1 being the most abundant mRNAs, and with the pan-reactive signature, with an increase of Lcn2 transcripts also involved in neurotoxicity. Wu et al. (2019) obtained similar results with astrocytes sorted from the hippocampus of TauP301S mice at 6-month-old of age. TauP301S astrocytes shared an induction of A1 and pan-reactive astrocyte genes, such as C3, Serping 1, H2-D1, Ggta1, and Ligp1. This induction was stronger than in forebrain astrocytes from PS2APP mice (7–13 months of age). The authors described a strong increase of C3-positive astrocytes in both models but with a more robust one in the TauP301S hippocampus. Sidoryk and colleagues have shown that primary astrocytes extracted from 7 day-old newborn TauP301S pups already exhibit defects in neuroprotective features, such as a reduction of thrombospondin-1 (TSP-1) expression (Sidoryk-Wegrzynowicz et al., 2017; Wu et al., 2019), that negatively impact synapses formation and cell survival (Christopherson et al., 2005).

The specific expression of T34 human tau isoform selectively in astrocytes in a transgenic mouse model led to astrocytic morphological alterations resembling those found in corticobasal degeneration (CBD) or other primary tauopathies (see the section “Pieces of Evidence of Astrocyte Neurotoxicity in NDD Patient Samples”) called tufted astrocytes, astrocytic plaques, or threads (Forman et al., 2005). However, no neuronal loss was observed in this model but focal neuronal injury and mild blood–brain barrier disruption. Richetin et al. (2020) showed that the overexpression of 3RTau in the hilus of mice *via* viral transduction led 4 months later to an impaired inhibitory circuitry and synchronous activity with a decrease of parvalbumin-positive neurons and vesicular GABA transporter (VGAT) positive synapses. The stimulation of parvalbumin-positive interneurons with neuregulin-1 peptide rescued the spatial memory impairments. In this model, the general neuritic density was not affected. However, the number of immature doublecortin-newborn neurons was reduced indicating a selective detrimental impact of tau-expressing astrocytes on neurogenesis.

To conclude, astrocytes are found detrimental in most models *in vitro* and *in vivo* when they react to oligomers, fibrils, and inclusions or carry Tau aggregates or Tau isoforms.

#### Synuclein Pathology

Exposure to monomers and preformed fibrils (PFFs) of α-Synuclein can also change astrocyte molecular profiles towards neurotoxic states. Chou et al. (2021) found that the treatment of human midbrain primary astrocytes by synuclein PFFs induced an nuclear factor kappa B (NF-κB)- and RIPK1-dependent inflammatory factor release; an A1 partial signature (SERPING1, HLA-E, SRGN, and PSMB8) impacted MEGF10 and MERTK activity. Chavarría et al. (2018) described concordant results after incubation of primary rat cortical astrocytes with monomers, oligomers, and PFF of α-Syn. Pre-treated astrocytes were found to engage neurotoxic features by drastically decreasing the survival of primary hippocampal neurons after 72 h, with a gradual effect from monomers to PFFs. The astrocyte-induced neurotoxicity was defined by a mitochondrial dysfunction, subsequent oxidative stress, and by an overproduction of typical pro-inflammatory cytokines TNF-α and IL-1β. However, Russ et al. (2021) showed that human iPSC-derived healthy astrocytes treated with α-Syn monomers and fibrillar polymorphs react differently than the ones treated with TNF-α and do not engage a pro-inflammatory response. In their experimental procedure, α-Syn fibrils-treated astrocytes showed dysfunctional mitochondria respiration and adopt an antigen-presenting phenotype, with an increase of expression of the human leukocyte antigen (HLA) genes encoding MHC I and II. Characterizing the response of astrocytes to α-Syn in rodent models is not straightforward as only a few models overexpressing α-Syn have been reported to mimic some PD pathological features. We think that 1-methyl-4-phenyl-1,2,3,6-tetrahydropyridine (MPTP), 6-hydroxydopamine (6-OHDA), paraquat, and rotenone are irrelevant for this discussion because neurotoxin-induced models can alter directly the glial cells and neurons bypassing a sequential pathological cascade linked to α-Syn. The selective expression of A53T α-Syn in astrocytes has surprisingly led to a more severe phenotype than the expression of A53T α-Syn by a neuronal promoter in transgenic mice (Gu et al., 2010). These animals showed general astrocyte reactivity with a dysmorphic appearance, even in the pre-symptomatic phase, concomitant with a high neuronal loss in the midbrain and spinal cord, where microglia were also found activated and agglomerated. Mice became paralytic after 7 weeks and quickly died afterwards. In conclusion, based on models *in vitro* and mouse models experiments *in vivo*, α-Syn could trigger either neurotoxic phenotypes or antigen-presenting phenotypes. An aggregation of α-Syn in astrocytes could severely negatively impact the pathological progression of the disease.

#### TDP-43 Pathology and ALS Models

Similar experimental approaches were undertaken to expose astrocytes to TDP-43 pathology. In Smethurst et al. (2020) insoluble material from the spinal cord of ALS patients was used to seed TDP-43 aggregation in transfected human IPSC-derived astrocytes. Phosphorylated TDP-43 inclusions were found but in lesser quantity than in human motor neurons after the same treatment. In co-culture experiments, healthy astrocytes exerted a protective function alleviating TPD-43 spreading and pathology. Transfecting rat primary astrocytes with a vector expressing the C-terminal fragment of TDP-43 led to the formation of TDP-43 typical inclusions. TDP-43 positives astrocytes showed increased lipid droplets and a differential response to noradrenaline (NA) revealing calcium and metabolic dysfunction (Velebit et al., 2020). When Lee et al. have transfected primary cortical mouse astrocytes with the human form of TDP-43, they found an increase of inflammatory factors (IL-6, IL-1β, LCN2, iNOS, or NF-κB) dependent on the Protein tyrosine phosphatase 1B (PTP1B; Lee et al., 2020). The neurons in culture treated with TDP-43 transfected astrocyte culture medium had lower survival rates partially corrected by PTP1B inhibition.

The selective expression of the mutated form of TDP-43 (M337V substitution) in astrocytes in transgenic rat led to a severe degenerative phenotype with a progressive loss of motor neurons and concomitant atrophy of skeletal muscles leading to paralysis (Tong et al., 2013). Along with the disease progression, authors reported a decline of GLAST and GLT1, and the upregulation of Lipocalin 2 (Lcn2) and Chi3L1, two neurotoxic factors in a dose-dependent manner on cortical neuronal culture cells (Bi et al., 2013; Huang et al., 2014). When the same mutated form of TDP-43 (M337V) was expressed in neurons, LCN2 was progressively up-regulated in astrocytes and found in CSF.

Several studies using hiPSC-derived astrocytes from ALS patients have reported cell-autonomous astrocytic dysfunction and proposed a consecutive loss of the astrocytic protective/homeostatic function and/or increased toxicity. The release of TNF-α, the reduction of astrocytic glutamate uptake or the decrease of antioxidants may then result in non-cell-autonomous damage and consecutive death of motor neurons (MNs; Haidet-Phillips et al., 2011; Madill et al., 2017; Kia et al., 2018; Birger et al., 2019). Intranuclear RNA foci have been observed not only in neurons but also in a small proportion of astrocytes in postmortem CNS tissue from C9-ALS patients (Lagier-Tourenne et al., 2013) as well as in iPSC-derived C9-mut astrocytes (Zhao et al., 2020). Mutations in C9orf72 are the most common cause of ALS. Subsequent transcriptomic analysis revealed almost 700 dysregulated genes in C9-mut astrocytes with increased expression of genes involved in ionotropic glutamate receptor signaling (GRIA1, GRIA4), complement activation, ribosomal subunit assembly (large and small), and nuclear RNA export. Reduced gene expression was observed in genes involved in cell adhesion (L1CAM, TSP1, and NTN1), synapse assembly (BDNF, NRG1, and THBS2), cell-to-cell signaling (GPC6), regulation of sodium ion transport (SLC8A1, ATP1B2, and NKAIN4), and potassium ion import (DLG1, ATP1B2; Zhao et al., 2020). Another transcriptome analysis of C9-mut astrocytes further revealed a senescent phenotype with 899 differently expressed genes compared to controls, including upregulation of TNFRSF10D, SERPINE1, SA β-gal, p21, CDKN1A, and PTDGS, as well as downregulation of CDKN2C, STMN1, and E2F1.

Thus, there are many mechanistic roads to impact astrocyte phenotypes and induce molecular and structural changes that will affect surrounding cells. There is a huge variability between models but clear proof of principle that astrocytes could lead to disease progression and turn toxic to neurons. This turn to detrimental features is also hypothesized to be directly associated with the activation of the microglia.

#### Interplay Between Astrocytes and Immune Cells: The Control of Neurotoxicity?

Astrocytes have an intimate relationship with immune cells, especially with microglia with who they collaborate to respond and clean the insult of the CNS (Bouvier and Murai, 2015; Matejuk and Ransohoff, 2020). Microglia appear central to disease progression in AD and other NDDs (Heneka, 2019). When microglia get activated in pathological contexts, they engage phagocytic functions, activate anti-inflammatory or pro-inflammatory responses to fulfil their immune function, and protect the tissue (Salter and Stevens, 2017). Microglia influence astrocytes directly. Indeed, astrocytes express key cytokine/chemokine receptors at their surface (IFN-R, TNF-R, IL-R, and TLR) that sense inflammatory factors released by immune cells. They also respond to such stimuli by adapting their molecular profile and turning reactive. Interestingly, the A1 signature is fully dependent on microglia activation and on their release of pro-inflammatory cytokines, such as IL-1α, TNF-α, and C1Q as seen after LPS stimulation. In microglia depleted mice (Csf1r^−/−^) astrocytes did not adopt the A1 signature after LPS injection. To further investigate microglia-astrocyte crosstalk *in vitro*, Guttikonda and colleagues developed human pluripotent stem cells (hPSC)-derived tri-culture system containing pure populations of hPSC-derived microglia, astrocytes, and neurons (harboring the APPSWE^+/+^ mutation or isogenic controls; Guttikonda et al., 2021). They found that C3 is increased in control tri-culture compared to astrocyte/neuron or neuron only cultures. In the APPSWE^+/+^ tri-cultures, C3 was further increased, but only in the presence of microglia. They identified microglial TNF-α as the main inducer of C3 expression in astrocytes but found that microglia were also releasing C3. Joshi et al. (2019) focused on the role of mitochondria damage in the induction of astrocytes neurotoxicity. They found a simultaneous reduction of microglia and astrocyte activation in mouse models of AD (5xFAD), HD (R6/2), and ALS (SOD1-G93A) by decreasing mitochondria fragmentation through the inhibition of the binding of the dynamin-related protein 1 (Drp1) to the mitochondria receptor Fis1 with the compound P110. They further reported that applying conditioned media of Q73 or mutant G93A SOD1 microglia (ALS model) showing excessive mitochondria damage, induced a similar mitochondria fragmentation in astrocytes and a concomitant A1 signature. Park et al. (2021) found that the astrocytic reactivity dependent of the microglia was associated with microglial Glucagon-like peptide-1 (GLP-1) receptor signaling. They showed that the GLP-1R was upregulated in 5xFAD mice. They have injected these mice with NLY01, an engineered agonist of GLP-1R, and observed suppression of Aβ-induced microglial activation and astrocytic reactivity as well as neuronal cell death, and partial rescue of cognitive decline. But microglia-astrocyte interplay is dynamic and astrocytes do influence microglia states as well. Rueda-Carrasco et al. (2021) showed that the astrocyte secreted frizzled-related protein 1 (SFRP1) is necessary for inducing microglia activation after LPS injections. McAlpine and colleagues found that IL-3 release by astrocytes is a regulator of microglia activation favoring their protective role against amyloid pathology in 5xFAD mice (McAlpine et al., 2021). They confirmed colocalization of IL-3 with astrocytes and IL-3R alpha with microglia in frontal cortex autopsy samples from AD patients. Neurotoxic astrocytes could also drastically alter microglia phenotypes. Indeed, C3 was found specifically expressed by astrocytes and C3aR by microglia in WT and APP/PS1 mice (Lian et al., 2016). The inhibition of the C3aR with an antagonist was sufficient to reduce amyloid pathology in APP/PS1 mice.

The relationship of astrocytes with the infiltration of peripheral immune cells, such as CD8 cells, in AD, and other NDDs, is still under scrutiny and would need to be further explored.

Gate and colleagues discovered that the presence of a subgroup of peripheral immune cells called the CD8^+^ T effector memory CD45RA^+^ or T_EMRA_ in individuals, after an analysis of AD patients, MCI individuals, and healthy controls, is predictive for cognitive decline (Gate et al., 2019). The T_EMRA_ were often found in the vicinity of Aβ blood vessels and astrocytes are good candidates to serve as chemoattractants for peripheral immune cells infiltration. Some cytokines and chemokines associated with neurotoxic signatures, such as IL-6, IL-1β, or CXCL10 have been involved in the blood brain barrier (BBB) disruption and the attraction of B and T cells (Rothhammer and Quintana, 2015).

The states of the astrocytes, beneficial or detrimental, are intermingled in a more global reaction of brain cells (Figure 1) and their bilateral interaction with the brain and peripheral immune cells is at the center of numerous short-term and long-term responses that could shape the progression of NDDs.

#### Depletion of Noradrenaline and Dopamine, Vectors for Neurotoxic Astrocytes?

In the most common NDDs, astrocytes, among other brain cells, are confronted with numerous global brain alterations. Apart from pathological protein inclusions, the brain is affected by a progressive alteration of its neurochemistry as well as an imbalance of numerous neurotransmitters that disturb the activity of neurons and glial cells. The progressive depletion of NA and dopamine (DA) is associated with ageing (Manaye et al., 1995; Volkow et al., 1998; Beardmore et al., 2021) and the onset of NDDs such as AD and PD (Weinshenker, 2018; Biondetti et al., 2021). A local change of availability of NA or DA could strongly alter the responses of astrocytes in NDDs and favor their neurotoxicity.

The degeneration of the locus coeruleus (LC), the primary source of NA, is a hallmark shared by multiple neurodegenerative disorders (Holland et al., 2021). The LC is a small brainstem nucleus mainly composed of NA producing neurons (Hansen, 2017) innervating multiple brain regions, such as the hippocampus, the amygdala, and the prefrontal cortex. NA is essential for hippocampus-based declarative memory formation, and for the regulation of cellular responses such as neuroinflammation and neuronal survival (Matchett et al., 2021). Its unbalance or progressive depletion in NDDs could impact the responses and fate of astrocytes. Indeed, astrocytes express numerous noradrenergic receptors at their surface (α and β), and NA modulates their metabolic activity, glutamate uptake, glycogen production, and glucose metabolism (O’Donnell et al., 2012), but also their calcium activity (Ding et al., 2013; Oe et al., 2020). NA has been also directly involved in astrocyte mediated memory consolidation (Gao et al., 2016; Wahis and Holt, 2021). NA can downregulate transcription of pro-inflammatory genes (such as TNF-α, IL-1β, and iNOS), and upregulate anti-inflammatory molecules (such as HSP-70 and MCP-1) in astrocytes and microglia (Heneka et al., 2010; Chalermpalanupap et al., 2013). The DSP4 (N-(2-chloroethyl)-N-ethyl-bromo-benzylamine) model is based on a systemic administration of the selective neurotoxin DSP4 that causes a huge NA depletion through a terminal retrograde degeneration of the majority of LC-noradrenergic neurons (Carnevale et al., 2007). In this model, astrocytes increased their IL-1β expression in response to NA depletion (Heneka et al., 2002). Heneka and colleagues showed with a DSP4-APP23 transgenic mouse model that astrocytes develop a reactive phenotype and express a plethora of pro-inflammatory molecules (Heneka et al., 2006). In this study, NA deficiency increased neuronal loss in CA1 hippocampus and frontal cortex, plaque loads, CD11^+^ microglia, GFAP, and iNOS expressions, and induced the formation of NO-mediated peroxynitrite, a free radical with high cell toxicity (Vodovotz et al., 1996; Smith et al., 1997). High-GFAP astrocytes were also shown highly abundant in the hippocampus after DSP4 treatment of an APP mouse model (9-month-old male mice) in concomitance with higher plaque loads (Kalinin et al., 2007).

Dopamine (DA) is an essential catecholamine and neurotransmitter (Meiser et al., 2013). Dopaminergic neurons are distributed in nine cell groups from the midbrain to the olfactory bulb. In adult brain, dopaminergic pathways project from the substantia nigra pars compacta (SNpc) to the striatum, and from the ventral tegmental (VTA) area to the cortex (Björklund and Dunnett, 2007). It forms three mains dopaminergic pathways: the nigrostriatal pathway, the mesolimbic pathway, and the corticolimbic pathway. In PD patients, the nigrostriatal pathway is severely affected because of the specific degeneration of dopaminergic neurons located in SNpc (Arias-Carrián et al., 2010). A decrease in the DA levels was also shown to be significant in dementia of Alzheimer type (Adolfsson et al., 1979). DA deprivation in PD and other NDDs could also impact the phenotypes of astrocytes in DA targeted areas. Indeed, astrocytes from various brain regions can express DA receptors at their surface, D1–D5 (Khan et al., 2001; Miyazaki et al., 2004; Xin et al., 2019). DA can directly affect their Ca2+ signaling. Nucleus accumbens astrocytes respond to DA through D1 receptors and seem to mediate DA-evoked depression at the synaptic level (Corkrum et al., 2020). Galloway et al. (2018) demonstrated an interesting role of DA in the epigenetic remodeling of primary astrocytes in culture. Both NA and DA deprivation could have a profound effect on astrocytes and prime them to maladaptive responses.

### Pieces of Evidence of Astrocyte Neurotoxicity in NDD Patient Samples

Neurotoxic states of astrocytes can be triggered by numerous stressors *in vitro*, in mouse and human cells as well as in rodent models. Direct exposure or internalization of NDD typical pathological protein inclusions, and/or unbalance of neurotransmitters/neuromodulators, activation of microglia are all factors involved in the triggering of astrocyte neurotoxicity. There is no consensus around a prototypical signature. However, some overlap was found in different models. Some studies also gave contradictory conclusions on the role of astrocytes in disease progression. As the experimental set-ups impact the responses of astrocytes, translational research is mandatory to further understand their roles in the neurodegenerative cascades. The rise of new technologies such as snRNAseq allows for investigating frozen human post-mortem tissue with a precision never obtained before. The published data already reflect the heterogeneity of their states across brain regions, stage of the disease, and condition. It is now essential to associate the state of an astrocyte to its macro- (type and stage of the disease, brain region) and micro-environment (pathological protein inclusion proximity, inflammation associated to microglia and peripheral immune cells) to further understand its involvement in disease progression. Few points to consider before extrapolating *in vitro* and rodent model data to the human brain:

- For each NDD, the concomitance of typical pathological inclusions is more the rule than the exception (Golde et al., 2013; Robinson et al., 2018).
- The ageing process is poorly mimicked in cell culture and transgenic rodents.
- Human astrocytes are distinct from rodent ones in many aspects and their heterogeneity is greater.

Indeed, human astrocytes are different from mouse ones in size, morphology, proportion per neuron, and responses (Oberheim et al., 2006, 2009; Li et al., 2021). The Li et al. (2021) study confirmed the differential responses of immunoisolated mouse (P1-P3) and human astrocytes (gestational week 17–20) under oxidative stress, hypoxia, inflammatory conditions, and viral infections. Murine and human astrocytes showed already significant differences in gene expression in serum-free conditions. These differences persisted through development even when human astrocytes were grafted in a mouse brain, attesting to an intrinsic differential program. Facing stress and pathological conditions, human astrocytes differed in their mitochondria-energy metabolism and immune responses showing more vulnerability to oxidative stress and capacity to engage the antigen presentation pathway. The same authors have identified a core astrocyte signature in human NDDs by comparing transcriptomic analysis of AD and MS cases to poly I: C-, and TNF-α-induced changes of cultured human astrocytes. Some commonly upregulated genes were associated with inflammation such as C3, IFITM3, NFkBIA, CCL2, and CXCL10 and have been associated with neurotoxic signatures.

In this chapter, we attempt to map out molecular signatures, markers, and pieces of evidence of astrocyte neurotoxic responses in NDD patient brains.

#### Alzheimer’s Disease

A high level of GFAP is commonly detected by positron emission tomography (PET; Carter et al., 2012, 2019; Verberk et al., 2020; Calsolaro et al., 2021; Chatterjee et al., 2021), in the blood (Goetzl et al., 2018; Cicognola et al., 2021) or CSF (Sathe et al., 2019) of MCI and AD patients. This GFAP level increase has been associated with the level of Aβ (Pereira et al., 2021) and negatively correlated with Mini-Mental State Examination score (Oeckl et al., 2019). The analysis of isolated astrocyte-derived exosomes (ADE) GLAST positive from plasma of AD patients and controls showed significantly higher levels of the cytokines IL-6, TNF-α, and IL-1β and of numerous complement proteins (Goetzl et al., 2018). Surprisingly, GFAP is also described as a potential biomarker for early AD in the saliva of MCI and AD patients where its levels are correlated with Aβ_42_, IL-1β, and caspase-8 (Katsipis et al., 2021). An increase of GFAP staining is commonly observed in the parenchyma of AD cases (Serrano-Pozo et al., 2013). Kobayashi and colleagues classified their cohort of samples into three groups, the control group, AD with and without dementia (Kobayashi et al., 2018). They found an increase of GFAP in the entorhinal cortex of both AD groups, however; the non-demented AD group was characterized by an increase in the expression of GLT-1. A recent analysis of astrocytes markers across IHC and RNAseq data collections in human samples have highlighted the complexity of astrocyte responses in AD and delimited a core reactive signature of AD astrocyte (ADRA) that points towards dysfunctions and neurotoxicity (Viejo et al., 2021). The authors build an online resource where each ADRA marker confirmation by IHC or RNAseq is mapped.

Numerous studies characterized astrocyte neurotoxic markers in post-mortem AD samples. C3-positive astrocytes were detected in numerous NDDs (Liddelow et al., 2017), in the entorhinal cortex layers I-III and CA1 hippocampus with a co-expression of serine racemase, an enzyme that produces D-Serine (Balu et al., 2019), and in the frontal upper cortex (King et al., 2020). Chun et al. (2020) found an increase of Nos2 in the AD temporal cortex. Subpial and RGN astrocytes expressed Nos2, Nos3, and nitrotyrosine in AD hippocampus, frontal, temporal, and entorhinal cortices (Heneka et al., 2001; Lüth et al., 2002). IL-6-positive astrocytes were detected in the lateral hypothalamus, cingulate cortex (Lyra e Silva et al., 2021), and prefrontal and temporal cortices (Bouvier et al., 2016). Recent snRNAseq data also gave new insights on astrocyte molecular status in AD. Grubman et al. (2019) found two AD specific astrocyte clusters from the entorhinal cortex of AD patients. One was defined by an increase of genes related to ribosomal, mitochondrial, neuron differentiation, and heat shock responses and the other cluster by enrichment for transcripts related to transforming growth factor (TGF)-β signaling and immune responses. Neither of them overlapped significantly with the A1 or A2 profiles but the second cluster showed an upregulation of C3. Lau et al. (2020) performed snRNAseq on AD prefrontal cortex and control samples. They have identified two “AD-upregulated” astrocytic subpopulations expressing CRYAB, GFAP, LINGO1, and HMGB1 and one “AD-downregulated” subpopulation. The authors concluded for dysregulation of neurotransmitter recycling and an exaggerated alarming response of astrocytes. Gerrits et al. (2021) proceeded to single-cell RNA-seq of the entorhinal cortex of AD patients, but they did not find any AD-associated changes in astrocytes.

Overall, there are no prototypical neurotoxic signatures such as the A1 or DAA ones found in AD yet. However, some subgroups of astrocytes showed some overlapping trends of transcripts or increase of markers associated with severe reactivity and/or neurotoxic responses.

#### Primary Tauopathies

Primary tauopathies are defined by a tau-driven pathology, the absence of Aβ plaques and are associated with presenile dementia. They are grouped according to their ratio in the predominant isoform, either the 3-repeat tau (3R) or the 4-repeat tau (4R) in 3R, 4R predominant, or mixed. They comprise 3R Pick disease, and 4R CBD, progressive supranuclear palsy (PSP), globular glial tauopathy (GGT), and argyrophilic grain disease (AGD; Chung et al., 2021). All these NDDs are characterized by the presence of tau-positive astrocytes reviewed in (Kovacs et al., 2016). The expression of tau by astrocytes, mostly 4R, directly impacts their morphologies. They are called tufted astrocytes, astrocytic plaques, ramified astrocytes, globular astroglial inclusions (GAIs), Thorn-shaped astrocytes (TSA), and granular/fuzzy astrocytes (GFA). The post-mortem stratification of patients in the “ageing-related tau astrogliopathy” (ARTAG) group is now based on the high frequency of TSA and GFA in specific anatomical regions (Kovacs et al., 2016, 2018; McCann et al., 2021). However, the relationship of tau positive astrocytes with neurodegeneration in human post-mortem NDD samples is still under scrutiny. Briel et al. (2021) analyzed the relationship between astrocyte plaques and tufted astrocytes and the peripheral synaptic density in cortical and striatal areas from PSP and CBD patient samples. General lower excitatory and inhibitory synapse densities were found significant in PSP Frontal Cortex. Local synaptic density was negatively correlated with astrocytic plaques only in CBD cases. Using image analysis, the authors also demonstrated a local loss of synapses in the territory of tau-positive astrocytes, especially exacerbated in astrocyte plaques. Interestingly tufted astrocytes were also found in Lewy body disease (Hishikawa et al., 2005) and were not associated with pathological inclusions but with age.

#### Parkinson’s Disease and Synucleinopathies

Synucleinopathies are characterized by the pathological accumulation of α-Syn mainly in neurons but also in glia, in the form of granular intra-cellular accumulations, Lewy neurites and Lewy bodies. The synucleinopathies are classified into three major entities: PD, LBDs which comprise Parkinson’s disease dementia (PDD) and dementia with Lewy bodies (DLB), and multiple system atrophy (MSA). DLB is the second most common cause of dementia and often share amyloid and tau pathologies with AD. In most of the synucleinopathies, astrocyte reactivity is considered as low or inexistent in the human brain, which rather contradicts previous conclusions made *in vitro* and mouse model studies. Mirza et al. (1999) did not observe differences in GFAP density, distribution or morphology of astrocytes in the substantia nigra (SN), or putamen in post-mortem samples from PD patients compared to age-matched controls. Tong and colleagues confirmed these results at the protein level by reporting similar levels of GFAP, Vimentin, and heat shock protein-27 (Hsp27) by quantitative immunoblotting in the SN from PD patients compared to controls (Tong et al., 2015). However, all these markers were increased in SN and putamen of MSA patients. Looking across LBD cohort samples, Van Den Berge et al. (2012) did not find any increase of astrocyte reactivity using GFAP and vimentin IHC or Western blot in the frontal cortex of PD, PDD, and DLB patients. Few reports found a change in the astrocyte signatures in PD and DLB. Rostami et al. (2020) reported the expression of MHC2 markers colocalized with GFAP staining nearby CD4^+^ cells in PD brains. Few “neurotoxic” markers were found in astrocytes in PD brains: C3 in SN and frontal cortex (Liddelow et al., 2017) and an increase of LCN2 in the SN of PD patients by Western blot analyses. Some TNF-α and iNOS positive astrocytes were detected in the hippocampus of DLB patients (Katsuse et al., 2003). Agarwal and collaborators performed snRNAseq on SN from PD and control subjects and found two PD astrocyte clusters, one with upregulated neuroinflammatory genes (Olr1), and one expressing gene associated with growth and reparative functions (Gins3; Agarwal et al., 2020). The authors do not report any A1, A2, pan-reactive, or other previously described reactive astrocyte signatures. However, astrocytic reactivity is found in the proximity of alpha-synuclein inclusions in the white matter of visual and frontal cortices and the grey matter of the putamen in MSA brain samples (Radford et al., 2015). Inoue et al. (2021) described a positive correlation of GFAP with the expression of the stimulator of interferon genes (STING) in the putamen and SN of MSA cases. STING, a cytosolic DNA sensor can trigger type 1 interferons and is involved in defensive immune mechanisms against pathogens but also in autoimmunity. Astrocytic toxicity across synucleinopathies remains uncertain but could be more prominent in MSA than in PD or LDB progression.

#### TDP-43 Associated Proteinopathies

Although ALS and FTD are clinically distinct NDDs they genetically and pathologically overlap and share central features (Gerovska et al., 2020). Around 30% of ALS patients and up to 15% of FTD patients will develop an overlap of clinical features (for review, see Lomen-Hoerth, 2011). Intraneuronal accumulation of misfolded, ubiquitinated/phosphorylated proteins, such as TDP-43, C9orf72 (C9), superoxide dismutase 1 (SOD1), and fused in sarcoma (FUS), is a major key factor in sporadic and familial ALS (sALS, fALS; for review, see Peters et al., 2015; Volk et al., 2018) but are not exclusive to ALS. Accumulation of TDP-43 can be found in the majority of cases of frontotemporal lobar degeneration (FTLD), so-called FTLD-TDP (Arai et al., 2006; Neumann et al., 2006) as well as in limbic-predominant age-related TDP-43 encephalopathy (LATE; Nelson et al., 2019). Astrocytes are hypothesized as significant actors in ALS and FTD progression mainly due to results from *in vitro* and *in vivo* rodent models (Izrael et al., 2020). Analyses of human post-mortem spinal cord tissue revealed enrichment of astrocyte-specific genes and enlarged perivascular spaces with separation of astrocyte and mural basement membranes in sALS (Månberg et al., 2021). RNA-seq datasets implicated enrichment of upregulated DEGs related to astrocyte functions in ALS compared to control spinal cord samples (Wang et al., 2021). Blood-spinal cord-barrier (BSCB) disruption and leakage has been described in ALS patients and detachment of astrocytic end feet from vessels (Miyazaki et al., 2011) or regional differences in astrogliosis or GFAP expression (Schiffer et al., 1996; Oberheim et al., 2009; Sofroniew, 2015) are proposed to be responsible for a reduction of GFAP in the perivascular space (Waters et al., 2021). Further research on post-mortem human brain and/or spinal cord tissue indicated astrocyte-related neurotoxicity and/or MN loss mediated. This is reflected by an increase of astrocytic cystine/glutamate antiporter (xCT) as a response to oxidative stress, a decrease of astrocytic glutamate transporter GLT-1 (Rothstein et al., 1995) further leading to increased extracellular glutamate accumulation (Kazama et al., 2020), an increase of astrocytic connexin 43 (Cx43; Almad et al., 2016), and an increase of astrocytic chitinase-3-like protein 1 (CHI3L1) and 2 (CHI3L2; Sanfilippo et al., 2017; Vu et al., 2020), the latter negatively correlated with the survival time of ALS patients. Gorter et al. (2019) reported the expression of small heat shock proteins (HSPBs) in reactive lateral column astrocytes in ALS patients with short disease duration (SDD; HSPB5, HSPB8) as well as with moderate disease duration (MDD: HSP16.2). HSPBs are required for protein quality control and are relevant for stabilization of intermediately folded proteins to prevent misfolding and/or aggregation and subsequent cytotoxicity (Sharma et al., 1997; Van Montfort et al., 2001; Jaya et al., 2009; Haslbeck et al., 2015) and HSPB8 facilitates autophagy *via* BAG3 interaction (Fuchs et al., 2010, 2015). Thus, the upregulation of HSPBs could be a direct response to altered protein homeostasis. The pathogenic protein aggregates may subsequently trigger the glial inflammasome. Microglial NLR family pyrin domain containing 3 (NLRP3) inflammasome activation is an emerging key factor of neuroinflammation and contributor to disease progression in an ALS mouse model (Deora et al., 2020) and may further contribute to disease progression during neurodegeneration. Inflammasome components, such as NLRP1, NLRP3, adaptor protein apoptosis-associated speck-like protein containing a CARD (ASC), and interferon-inducible protein AIM2 (AIM2) colocalize with GFAP positive astrocytes in spinal cords of sALS patients (Johann et al., 2015; Hummel et al., 2021). Taken together, astrocyte-associated toxicity and neuroinflammation could be significant factors in the progression of ALS-related neurodegeneration.

## The Impact of Ageing on the Responses and Phenotypes of Astrocytes in NDDs

It is interesting to note that in most of the experimental models, the highest risk factor for NDDs which is ageing is strongly neglected. However, enough studies have demonstrated the change of states of astrocytes across ageing and their tendency to mimic certain reactive and even neurotoxic features. The cellular senescence is also an important factor to consider as senescent astrocytes would be detrimental for their surroundings.

### Ageing Signatures: Does the Progressive Loss of Homeostatic Astrocytic Phenotypes Prime Neurotoxicity?

In the ageing CNS, cells that are in mitosis or proliferating represent a small minority. Pools of new neurons, and sometimes astrocytes, are born from the neural stem cells of the subgranular zone (SGZ) in the dentate gyrus (DG) of the hippocampus and subventricular zone (SVG) but decrease in ageing mice (Baptista and Andrade, 2018). In humans, neurogenesis in the adult and ageing brain is still difficult to assess and results in the literature are often contradictory (Sorrells et al., 2018). However, Boldrini et al. (2018) showed that neurogenesis is relatively stable during ageing in DG and at least persists in humans until their 80s. Astrocytes seem to have a long lifespan with a low proliferation rate limited to severe astrogliosis in acute injuries or advanced stages of NDDs (Colodner et al., 2005; Sofroniew and Vinters, 2010). New astrocytes can be occasionally produced by the stem cells of SGZ (Bonzano et al., 2018) but the absence of data collected from the human brain does not allow further discussion. The general agreement is that astrocytes also show stereotypical phenotypic changes in ageing.

Numerous analyses based on GFAP and vimentin staining quantification in brain sections showed their increase in ageing in frontal, temporal, and entorhinal cortices, in the hippocampus of rats (Nichols et al., 1993; Amenta et al., 1998; Bernal and Peterson, 2011) and humans (David et al., 1997; Porchet et al., 2003). No change was observed in the chimpanzee brain (Munger et al., 2019). Cerbai et al. (2012) reported smaller astrocytes with simplified arborization in CA1 Stratum radiatum of aged rats compared to adults. Bronzuoli et al. (2019) showed by Western blot and immunofluorescence a reduction of GFAP, S100B, and connexin-43 expression but an increase of aquaporin-4 in hippocampal astrocytes from 12-month compared to 6-month-old mice. Thus, the homeostatic functions of astrocytes could be progressively altered during ageing. By using the Ribo-Tag technique, Boisvert et al. (2018) isolated specifically the ribosomal RNA of mouse astrocytes at specific ages from the visual (VC), the motor cortex (MC), hypothalamus (HTH), and cerebellum (CB). By comparing 2-year-old mice transcriptomes to 4 months old, they found that seven genes are commonly upregulated in all regions isolated, which are the serine protease inhibitor A3N and M (Serpina3n and Serpina3m), Gfap, some proto-cadherins-b 6 and 11, and C4b a component of the complement cascade. Some transcripts increased were specific to astrocytes of certain regions, such as Bmp6 and Sparc in VC astrocytes and pro-inflammatory factors such as Cxcl5, caspase-1 and 12 along Tlr-2 and 4 in CB. Overall, astrocytes seem to engage an ageing functional decline that could disrupt their interplay with surrounding neurons and alter their response to stress and pathology in older individuals. In line with these observations, Clarke and colleagues described a prevalence of the A1 or neurotoxic signature in astrocytes of older mice (Clarke et al., 2018). With a similar strategy to the Boisvert study, a translating ribosome affinity purification (TRAP) technique was used to isolate RNA astrocytes from the hippocampus, cortex, and striatum, later analyzed by RNAseq, across the lifespan of a mouse (Boisvert et al., 2018). They showed that aged astrocytes upregulate a high number of the A1 genes, especially in the hippocampus and striatum. Serpina3n, complement (C3 and C4B), and cytokine pathway (Cxcl10), but also antigen presentation (H2-D1 and H2-K1) were some of the most prominent. In parallel, the downregulation of genes involved in metabolic functions, such as mitochondria energy production and antioxidant defense, pointed out a general decline of astrocyte homeostasis. Upregulation of genes involved in synaptic elimination and extracellular matrix degradation was confirmed in mouse aged isolated- astrocytes by another study (Pan et al., 2020). Interestingly, Habib et al. (2020) found a strong association between the DAA signature and aged astrocytes in mouse wild-type and healthy humans. Soreq et al. (2017) have highlighted the severe impact of ageing on glial cells and more specifically on astrocytes and oligodendrocytes through an extensive analysis of gene expression datasets of numerous brain regions coming from postmortem tissue of 480 individuals between 16 and 106 years old. They were able to report a shift of identity of hippocampal astrocytes, more related to cortical ones in young humans, toward intralobular white matter and putamen astrocytes in the aged brain. Natural and healthy ageing is then a significant factor in the ability of astrocytes to maintain their homeostatic functions and respond to insults. The overlap of signatures between human astrocytes of aged individuals and A1 or DAA neurotoxic ones is intriguing and could contribute to their maladaptive or detrimental responses in the early phases of NDDs.

### Cellular Senescence in Astrocytes: The Ultimate Fate?

Cellular senescence is a hallmark of ageing. It is characterized by the irreversible loss of the ability of the cell to divide. It is a direct consequence of telomeres shortening and comes as a protective mechanism to avoid genome instability and cancer cell production (Di Micco et al., 2021). Senescent cells are not restricted to aged CNS, but their proportion is likely to increase during the ageing course and could affect or trigger some pathological cascades associated with NDDs (Rueda-Carrasco et al., 2021). Senescent cells can be identified by measuring the increased expression of cell cycle inhibitory proteins such the cyclin-dependent kinase inhibitors p21 and p16^INKA4^, the tumor suppressor protein p53, and by the ectopic expression of the senescence-associated beta-galactosidase (SA-β-Gal). Cellular senescence also induces a general change of the molecular state of the cell that provokes morphological alteration, metabolic stress, and chromatin remodeling. Cells under the senescent-associated secretory phenotype (SASP) secrete proinflammatory molecules, metalloproteinases, and growth-stimulating factors (Swenson et al., 2019). Senescent cells are resilient to cell death and are often called “zombie” cells. They can create local chronic inflammations in the CNS parenchyma and destabilize the micro-environment. Baker and collaborators demonstrated the impact of senescent cells *in vivo*, in healthy and aged cells (Baker et al., 2016). The authors have shown the ablation of senescent cells promotes normal tissue function and delays the onset of age-related pathologies. The same effects have been observed in mice (Zhu et al., 2016). Cellular senescence features partially overlap with neurotoxic astrocytes ones, which challenges their identification in the astrocytic population. *In vitro*, multiple conditions and stressors can activate a senescence program in astrocytes. Ageing can be mimicked in a dish by multiplying the number of passages of cells, by cultivating them over a longer period or by using primary cells extracted from aged animals. All these conditions trigger senescence in cultivated microglia and astrocytes (Bitto et al., 2010; Caldeira et al., 2014; Stojiljkovic et al., 2019). Many chemicals or active molecules have been used *in vitro* as stressors to induce senescent-like states in mouse or human astrocytes (Bitto et al., 2010; Stojiljkovic et al., 2019). Hydrogen peroxide (Bitto et al., 2010) inflammatory challenges, such as repeated LPS exposure (Yu et al., 2012), irradiation (Limbad et al., 2020), transient oxidative stress (Crowe et al., 2016), Aβ (Bhat et al., 2012; Zhang et al., 2019), or even the herbicide paraquat (Chinta et al., 2018) can trigger senescence in astrocytes. Transcriptome analysis of oxidative stress-induced senescence in human fetal astrocytes in culture (Crowe et al., 2016) revealed an upregulation of genes associated with inflammation and extracellular remodeling and a downregulation of genes involved in cell cycle and of GFAP and S100B. The senescence accelerated mouse (SAM) strain induces a shortened life span, loss of normal behavior, senile amyloidosis, and mitochondrial dysfunction, deficits in learning and memory, and brain atrophy (Takeda et al., 1997). Interestingly, SAM isolated astrocytes are more sensitive to artificial oxidative stress mimicked by an H_2_O_2_ exposure (Lü et al., 2008). Additionally, senescence has been shown to modify astrocytic ROS detoxification responses (Lü et al., 2008) and to compromise glutamate and potassium transport by decreasing the expression of EAAT1, EAAT2, and Kir4.1 (Limbad et al., 2020). Thus, *in vitro* experiments have demonstrated that numerous stressors, usually associated with ageing or disease context, can activate senescence in astrocytes. However, if glial senescence occurs *in situ* still needs to be further investigated.

Astrocytes are stable cells with a low turnover and are theoretically more prone to senescence. However, the measurement of telomere shortening in astrocytes *in vitro* contradicts this idea (Flanary and Streit, 2004; Szebeni et al., 2014). In female mice, the proportion of hypothalamic senescent astrocytes increased with age and appeared to be mainly modulated by the ovarian estradiol, which would associate astrocyte senescence with an early reproductive decline (Dai et al., 2020). Bussian and colleagues measured an increase of the expression of SA-βGal in cells identified as microglia and astrocytes by transmission electron microscopy in MAPTP301SPS19 mice (Bussian et al., 2018). In this model, senescence in glia was not linked to ageing (6-month-old animals) but mainly to the tau neuropathology. Interestingly, treating astrocyte senescence had major outcomes in mouse models. The clearance of glia senescent cells using INK-ATTAC transgenic mice had multiple beneficial effects. It prevented gliosis, decreased NFTs deposition and degeneration of neurons, and helped to preserve cognitive functions. Xu et al. (2021) reported downregulation of the Yes-associated protein (YAP) paralleled with a decrease of lamin B1 in hippocampal astrocytes during normal ageing and in APP/PS1 mice. The downregulation of YAP was also shown in D-galactose and paraquat-induced senescent astrocytes. YAP is involved in cell proliferation, differentiation, and tissue regeneration. Its activation delays senescence *in vitro* and reduces cognitive defects in old AD-like mice. Gaikwad et al. (2021) described a high proportion of p16^INK4A^ astrocytes that were also positive for pathological tau oligomers (TauO) in the frontal cortex of AD and FTD patients. They found that TauO exposure induced the nuclear translocation and release of high mobility group box 1 (HMGB1) in primary astrocytes and consequent paracrine induction of senescent profile in culture. Preventing HMGB1 release using inhibitors over 8 weeks showed a significant decrease of p16^INK4A^ astrocytes in 12-month-old hTau mice, a reduction of tau pathology and neuronal loss, and partial rescue of cognitive defects. A high proportion of p16^INK4A^-positive astrocytes were found in FFPE post-mortem brain samples of 78–90-year-old healthy humans (Bhat et al., 2012). Their number was even increased in AD patients, with a respective average of 35 and 50%. This staining was correlated with an increase of metalloproteinase MMP-1 expression. If these numbers stand true across NDDs, senescent astrocytes may represent the main neurotoxic astrocyte population, drive the most transformative alterations in ageing, and exacerbate disease progression in NDD. Paradoxically, treating them may be more straightforward. However, senescent astrocytes will need to be carefully quantified with multiple markers across NDD to define if senolytic targeted treatment may become a serious curative opportunity.

### Epigenetic of Ageing and Ageing Astrocytes

Epigenetics is an emerging field in both diagnostics and research. It encompasses several distinct modifications of the DNA that impact transcriptional activity while leaving the nucleotide sequence unchanged (Gibney and Nolan, 2010). It includes methylation, histone modification, or non-coding RNAs (ncRNA). In general, a hypermethylated phenotype is associated with ageing processes in mammals. However, data about cell-type-specific epigenetic alterations were lacking for a long time (Maegawa et al., 2010). For brain tissue, differentially methylated sites (DMS) significantly associated with ageing were mainly localized within CpG islands and displayed a frequent hypomethylated status. Furthermore, age-associated DMS in brain tissue were enriched in H3K27me3 and frequently found within laminar-associated domains (LADs). H3K27me3 constitutes a repressive post-translational histone mark and LADs show general low transcription levels indicating a reduced gene activity in line with the ageing processes (Lochs et al., 2019). NDDs studies have shown significant differences in epigenetic marks, such as increased DNA methylation and hydroxymethylation in AD, features that correlated with pathological Aβ, tau, and ubiquitin load (Coppieters et al., 2014). A recent meta-analysis study about brain methylation revealed a very high correlation between age-related DNA methylation patterns in normal ageing and AD brain tissue, for both usually hypo- but with some hypermethylated CpGs (Pellegrini et al., 2021). It might indicate that NDDs may also be the result of abnormal, accelerated ageing processes. However, those epigenetic marks were differentially regulated between neuronal and glial cells, thereby indicating a rather cell type-specific epigenetic regulation in age-related CNS pathologies (Coppieters et al., 2014). Of note, mainly astrocytes and pyramidal neurons displayed an altered methylation profile while cell types that seem to be less affected in AD such as calretinin-positive interneurons remained relatively unaffected (Phipps et al., 2016). To better understand the implication of epigenetic regulation in the ageing process of astrocytes, it is important to understand the impact of epigenetics on the normal development of astrocytic cells. A pioneering study in the field showed that genes, that are generally considered as being astrocytic-specific, become rather de- or hypomethylated once neural precursor cells (NPC) start to differentiate into astrocytes (Hatada et al., 2008). This process allows key signal transducers in astrocytes such as SMAD or STAT3 to finally bind to their respective binding sites within astrocyte-specific genes such as GFAP, showing increased expression levels in both brain ageing and neurodegenerative disorders (Nichols et al., 1993; Porchet et al., 2003).

Epigenetic changes of the ageing astrocyte are still poorly understood. A murine stroke study revealed that aged astrocytes displayed less active chromatin, represented by weaker trimethylation of histone 3 lysine 4 (H3K4me3) acting as an enhancer while displaying stronger trimethylation of the repressive lysine residue at histone 3 lysine 9 (H3K9me3; Chisholm et al., 2015). This age-dependent impairment of active astrocytic gene transcription was associated with an increased stroke size. It indicates that aged astrocytes may be less capable of counteracting pathological conditions. Furthermore, it was demonstrated that inhibition of histone deacetylases (HDAC) increased the release of neurotrophic factors in astrocytes (Chen et al., 2006). It was shown in cell culture models that the administration of HDAC inhibitors led to increased clusterin levels, a molecular chaperone that may prevent disease progression in AD (Nuutinen et al., 2010). Although the first therapeutic strategies using HDAC inhibitors have been successful in AD-like mouse models (Francis et al., 2009) its translation into a clinical trial for NDDs seems to be much more challenging, as HDAC inhibitors may also have neurotoxic side effects (see review Shukla and Tekwani, 2020). In summary, epigenetic modifications play a central role in ageing processes, but it becomes more and more evident that these age-dependent changes or neurodegenerative alterations considerably differ between cell types. However, to date, it is still unclear if an epigenetically regulated cellular “age clock” can be selectively reverted to restore neuroprotective properties in a complex system such as the human brain, or if any interference with such fine-tuned, age-dependent epigenetic processes rather aggravates neurotoxic features in general or more specifically in astrocytes. Understanding the epigenetic regulation of astrocytes will be an asset to control their phenotypes in ageing and NDDs and may be important for the future development of novel treatment.

## Conclusion

**Figure 2:**
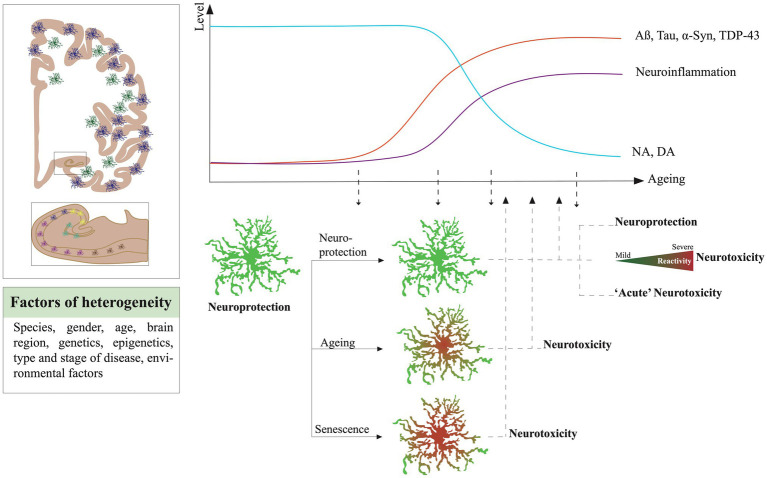
Hypothetical roads to astrocyte neurotoxicity in the context of neurodegenerative diseases (NDDs) and ageing. The heterogeneity of astrocyte response and change depends on numerous factors, such as the species, gender, age, anatomic localization, genetics and epigenetics, their micro-environment, and the type and stage of the disease. In NDDs, one or several subgroups of astrocytes could lose their neuroprotective function and become neurotoxic. Many factors could impact their transformation into toxic cells. The effect of ageing could prime them to maladaptive responses, cellular senescence would turn them detrimental to their surroundings, pathological protein inclusions [Aβ, tau, α-synuclein (α-Syn), and TAR DNA-binding protein 43 (TDP-43)], neuroinflammation and neurotransmitter concentration, and activation of immune cells would condition their shift to toxic thereby enhancing their detrimental features. Neurotoxic astrocytes would then contribute disease progression.

Many roads are leading to astrocytic neurotoxicity (Figure 2). There is a consensus about the potential of such an astrocytic phenotype to alter its micro-environment and exacerbate NDD pathologies and in the last few years, many researchers attempted to define a prototypical signature of a neurotoxic astrocyte (Figure 1). But what emerged from this translational review is a complex patchwork of heterogeneous neurotoxic signatures that vary across experimental models and conditions and are still controversial in human NDDs. Because cell culture or mouse models fail to fully recapitulate human NDDs and microscopy as well as even scRNAseq experiments from human samples provide only snapshots on the states of astrocytes, there is a need for more translational and multi-disciplinary approaches to solve the mystery of the roles of astrocytes in NDD progression. To advance in our understanding, astrocytic signatures in NDDs will have to be carefully associated with neuropathological diagnostics, especially focusing on distinct brain regions and composition of their surrounding microenvironment. There is still a lot to investigate before defining astrocytes as a therapeutic target in patients. Translational AD research points toward a strong evidence of direct implications of neurotoxic or detrimental astrocytes in the disease progression. However, their roles in other NDDs such as PD and ALS are still speculative and mainly based on *in vitro* and rodent model studies.

Furthermore, if we succeed in proving that neurotoxic astrocytes are leading neurodegenerative cascades, how could we treat them? Who to treat first, microglia or astrocytes? Both together? Should we target the ageing and senescence process or be more specific to the condition?

Non-targeted anti-inflammatory treatments (e.g., dexamethasone, minocycline, ibuprofen, and IL-10) have been efficient in animal models and have the benefit to target both microglia and astrocytes. But the search for an effective anti-inflammatory molecule for AD and NDD patients is still ongoing (Kwon and Koh, 2020). Non-steroidal anti-inflammatory drugs, such as ibuprofen, celecoxib, rofecoxib, or tarenflurbil have all failed to reduce cognitive decline in clinical trials in AD patients (Zanetti et al., 2009; Rivers-Auty et al., 2020).

The Nrf2 signaling pathway represents a potential target for the treatment of NDDs. Indeed, Nrf2 is a key endogenous regulator of oxidative stress and neuroinflammation (Johnson et al., 2008; Buendia et al., 2016; Sivandzade et al., 2019). Its overexpression prevented neurotoxicity in murine microglia and astrocytes (Sigfridsson et al., 2018; Heurtaux et al., 2021) but also protected neurons from potentially toxic insults (Shih et al., 2003; Bell and Robinson, 2011; Cui et al., 2016). Furthermore, pharmacological activators of Nrf2 are already in clinical development for treating traumatic brain injury, stroke, and cancer and could also be tested in NDDs (Robledinos-Antón et al., 2019; Zhou et al., 2020a).

Targeting senescence is also a promising avenue. Senotherapies target senescent cells to kill them or block their detrimental effects, e.g., growth arrest and the onset of a SASP (von Kobbe, 2019; Birch and Gil, 2020). Some senolytic cocktails relieved age-related brain inflammation and improved brain function in mouse models of NDDs (Ogrodnik et al., 2021). Finally, epigenetic manipulation is a promising approach to modulate the state of brain cells and reverse ageing and senescence. Over the last years, potential epigenetic inhibitors have emerged (Verma and Kumar, 2018). DNA methylation inhibitors (Decitabine, Zebularine, and 5’Aza) but also histone deacetylase inhibitors (Trichostatin A, Vorinostat) have shown promising results in experimental models of NDDs (Narayan and Dragunow, 2010; Konsoula and Barile, 2012; Coppedè, 2014; Kim and Hong, 2014). The use of genome-engineering tools, e.g., the CRISPR/Cas9 system, could also emerge as potential epigenetic regulators to treat patients (Yao et al., 2015; Vojta et al., 2016).

Overall, maintaining protective astrocyte function or alleviating astrocyte neurotoxicity seems a promising avenue for AD and NDDs. However, before crystallizing such a strategy, it is crucial to map with more precision the detrimental phenotypes of astrocytes across ageing and age-associated NDDs.

## Author Contributions

DB, SF, TH, FJ, KF, and MM performed literature searches and wrote the manuscript. DB build the structure of the manuscript and made the figures with SF. All authors contributed to the article and approved the submitted version.

## Funding

DB was supported by the Luxembourg Espoir-en-Tête Rotary Club award, the Auguste et Simone Prévot foundation, and the Agaajani family donation. SF was supported by the PRIDE program of the Luxembourg National Research Found through the grant PRIDE17/12244779/PARK-QC, and FJ was supported by the AFR FNR program. MM would like to thank the Luxembourg National Research Fond (FNR) for the support (FNR PEARL P16/BM/11192868 grant).

## Conflict of Interest

The authors declare that the research was conducted in the absence of any commercial or financial relationships that could be construed as a potential conflict of interest.

## Publisher’s Note

All claims expressed in this article are solely those of the authors and do not necessarily represent those of their affiliated organizations, or those of the publisher, the editors and the reviewers. Any product that may be evaluated in this article, or claim that may be made by its manufacturer, is not guaranteed or endorsed by the publisher.
